# First comprehensive case report of surgical resection of a cardiac Merkel cell carcinoma metastasis

**DOI:** 10.1016/j.xjtc.2025.07.011

**Published:** 2025-07-23

**Authors:** Christian Burgard, Willem Hendrik te Gussinklo, Thomas Ritz, Sameer Al-Maisary

**Affiliations:** a Department of Cardiac Surgery, University Hospital Heidelberg, Heidelberg, Germany; b Institute of Pathology, University Hospital Heidelberg, Heidelberg, Germany

**Figure:**
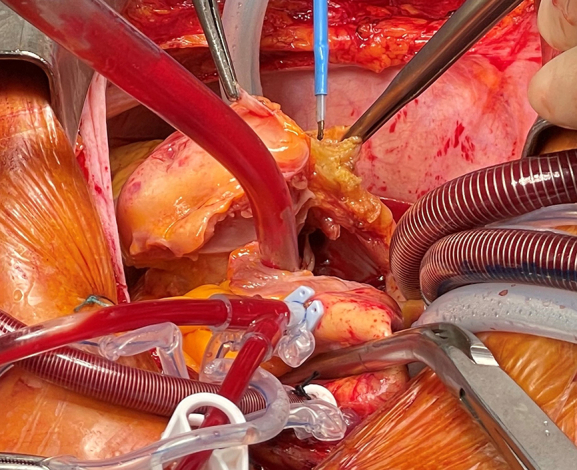
Merkel cell carcinoma metastasis infiltrating the right atrium and ventricle.

**Central Message**

Surgical resection of cardiac Merkel cell carcinoma metastasis is feasible and provides novel insights into therapeutic strategies, expanding the current knowledge of cardiac oncologic surgery.

Merkel cell carcinoma (MCC) is a primary cutaneous neuroendocrine tumor caused mainly by Merkel cell polyomavirus (∼80%) or ultraviolet-induced point mutations from sun exposure (∼20%). Distant metastases develop within 2 years of initial diagnosis in approximately 20% of patients. The most common distant metastatic manifestations are nonregional lymph nodes (41%), skin (25%), liver (23%), and bone (21%). Cardiac metastases in MCC are very rare, occurring in roughly 1.5% of metastatic MCC cases with limited experience, and usually are detected late in the disease course.^1^

Here we report the first comprehensively described case of a patient who underwent surgical resection of cardiac MCC metastasis. Written informed consent was obtained from the patient for publication of this case report and accompanying images. In accordance with institutional policy at the University Hospital Heidelberg, formal Ethics Committee approval was not required for this single case report.

## Case Description

A 64-year-old male with hypertension, dyslipidemia, and a history of MCC (initially diagnosed in 2020) was referred after detection of an incidental right atrial mass on routine imaging. The patient had undergone R1 resection of the primary skin tumor in 2020, followed by R0 re-excision. Later that year, a solitary bone metastasis in the ilium was treated with cumulative 45 Gy radiotherapy. From October 2020 to October 2022, he received immunotherapy with avelumab and denosumab, with stable restaging.

**Figure 1:**
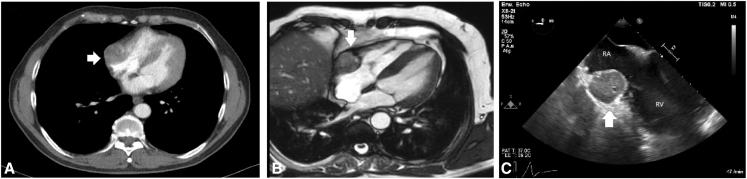
Preoperative chest computed tomography scan (A) and magnetic resonance imaging (B) and intraoperative transesophageal echocardiography (C) demonstrating a 3.0 × 3.7 × 4.0 cm mass involving the right atrium (RA), ventricle (RV), and lateral tricuspid annulus. The *white arrow* shows the tumor.

In July 2024, a computed tomography scan of the chest and abdomen revealed a 3.0 cm × 3.7 cm × 4.0 cm right atrial lesion in an asymptomatic patient (Figure 1). Transesophageal echocardiography (TEE) confirmed the mass; the differential diagnosis included metastasis, thrombus, and primary cardiac tumor. Cardiac magnetic resonance imaging further characterized the lesion, and the patient was referred for surgical resection.

The tumor was approached via median sternotomy, and bicaval cardiopulmonary bypass was initiated with cannulation of the ascending aorta in mild hypothermia (34 °C). Subsequently, the tumor was resected en bloc up to the tricuspid valve annulus, including the medial right coronary artery (RCA). The RCA stumps were clipped, and the anterior tricuspid leaflets were sutured to the right ventricular free wall. Right atrial reconstruction was done using a 5 × 10 cm bovine pericardial patch, and tricuspid annuloplasty was performed with a 30-mm Carpentier-Edwards Physio II ring (Edwards Lifesciences). A saphenous vein graft was anastomosed to the RCA. Cardiopulmonary bypass time was 150 minutes, and cross-clamp time was 126 minutes. Intraoperative TEE confirmed preserved right ventricular function without tricuspid valve regurgitation.

**Figure 2:**
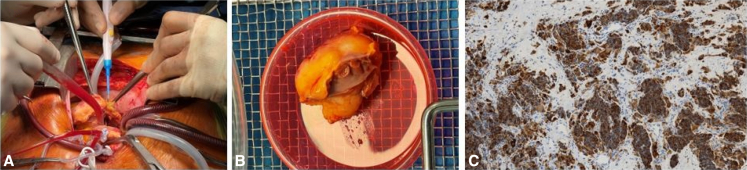
Intraoperative findings and histopathology. A and B, Tumor in the right atrium involving the right coronary artery. C, Cells with homogeneous positivity for the neuroendocrine marker chromogranin-A.

The tumor was positive for chromogranin-A (Figure 2), synaptophysin, and CK20 and had a significantly increased Ki-67 proliferation index. The tumor was removed with clear margins showing formation of a malignant, invasive, proliferative, small blue round cell neoplasia with multifocal lymphangioinvasion (L1) compatible with MCC.

The patient's postoperative course was uncomplicated, and he was transferred to the regular ward on postoperative day one and discharged on postoperative day 11 with stable sinus rhythm. Adjuvant avelumab immunotherapy was resumed. At a 3-month follow-up, TEE and cardiac computed tomography revealed a lesion adjacent to the right atrium consistent with either tumor recurrence or a postoperative scar. Further imaging identified new lymph node metastases. The patient completed 6 cycles of avelumab and was initiated on oral etoposide for stage IV MCC. At the time of this report, he remains under ongoing follow-up and treatment in cardiology and oncology.

## Discussion

Surgical resection of cardiac MCC metastases is very rare, with only 1 previous imaging report describing this approach.^2^ Case reports and small case series described patients receiving chemotherapy, immunotherapy, or radiotherapy, yielding a survival of 40% to 44%.^1^^,^^3^

Given the aggressive nature of MCC and the high risk of recurrence, careful patient selection and multidisciplinary decision making are essential. Our case demonstrates that complete resection with reconstruction is technically feasible, but short-term recurrence highlights the need for vigilant follow-up and adjuvant therapy. Future studies should clarify the role of surgery within a multimodal treatment strategy for cardiac MCC metastases, including whether surgical resection is a viable option for this type of entity and whether preoperative chemotherapy, immunotherapy, or radiotherapy may help prevent local recurrence.

## Conflict of Interest Statement

The authors reported no conflicts of interest.

The *Journal* policy requires editors and reviewers to disclose conflicts of interest and to decline handling or reviewing manuscripts for which they may have a conflict of interest. The editors and reviewers of this article have no conflicts of interest.
